# NO ONE REMEMBERS MY NAME: (RE)NEGOTIATING IDENTITY, AGENCY, AND RESILIENCE IN LATE-LIFE IMMIGRANT SOUTH ASIAN WOMEN

**DOI:** 10.1093/geroni/igae098.0213

**Published:** 2024-12-31

**Authors:** Mushira Khan

**Affiliations:** Mather Institute, Evanston, Illinois, United States

## Abstract

Despite a growing South Asian (SA) population in the US, little is known about the lived experiences of late-life SA immigrant women who may face unique challenges as they navigate their later years in a new cultural and social space. This qualitative study explored how late-life SA immigrant women negotiate issues around identity, faith, and the continuity/rupture of norms and familial bonds in the context of migration and multigenerational living. Using an intersectional lifecourse perspective, in-depth interviews were conducted with twelve SA women who immigrated to the US at age 65 or older and co-resided with their adult children and/or grandchildren in a large cosmopolitan US city. Thematic analysis of the interview data uncovered the resilience and strength of the study participants as they navigated settlement challenges, including culture shock, language barriers, health and social care access issues, and the intricate dynamics of multigenerational living. Drawing from their cultural heritage, they navigated these transitions while also (re)interpreting cultural traditions to find meaning and cope with a perceived loss of status in the family. Furthermore, despite migration challenges, study participants found solace in embracing their grandmother role and through support from their religious and cultural enclaves. These findings highlight the salience of faith, family, and community in the lives of late-life immigrant SA women–a group that often remains invisible in policy and practice–and can help policymakers and stakeholders, including community service providers, faith leaders, researchers, and family members, to provide culturally appropriate support and promote their health and well-being.

